# HPLC-MS identification and expression of *Candida* drug-resistance proteins from African HIV-infected patients

**DOI:** 10.3934/microbiol.2021020

**Published:** 2021-09-10

**Authors:** Pedro M D S Abrantes, Randall Fisher, Patrick J D Bouic, Carole P McArthur, Burtram C Fielding, Charlene W J Africa

**Affiliations:** 1 Maternal Endogenous Infections Studies (MEnIS) Research Laboratories, Department of Medical Biosciences, University of the Western Cape, Private Bag X17, Bellville 7535, South Africa; 2 Separated Sector Cyclotron Laboratory, iThemba LABS, Radiation Biophysics Division, National Research Foundation, Cape Town, South Africa; 3 Synexa Life Sciences, PO Box 36596, Chempet 7442, South Africa; 4 Department of Oral and Craniofacial Sciences, School of Dentistry, University of Missouri-Kansas City, MO 64108, USA; 5 Molecular Biology and Virology Laboratory, Department of Medical Biosciences, University of the Western Cape, Private Bag X17, Bellville 7535, South Africa

**Keywords:** *Candida*, drug resistance, HPLC-MS, protein expression, HIV

## Abstract

The objective of this study was to elucidate the proteomic mechanisms of drug resistance in HIV-infected African patients. Cell membrane fractions from forty oral *Candida* isolates isolated from African HIV-positive patients were analysed using HPLC-MS with the aim of identifying proteins associated with their pathogenicity and drug resistance. Heat shock proteins that mediate the fungicidal activity of salivary peptides were found in all tested *Candida* fractions, with pH-responsive proteins associated with increased pathogenicity only being present in the three most commonly isolated species. ABC multidrug transporter efflux pumps and estrogen binding proteins were only found in *C. albicans* fractions, while ergosterol biosynthesis proteins were identified in four species. The combination of various adherence, invasion, upregulation and efflux pump mechanisms appear to be instrumental for the *Candida* host colonization and drug resistance emergence in HIV-infected individuals.

## 1. Introduction

The opportunistic yeast, *Candida albicans*, can cause life-threatening infections with mortality rates exceeding 40% in treated cases [1], paralleled by a simultaneous increase in the numbers of resistant non-albicans *Candida* species [2]. Oropharyngeal candidiasis (OC) is diagnosed clinically by the appearance of white, plaque-like lesions of the tongue or oropharyngeal mucosa, usually confirmed by laboratory culture. As an early indicator of immune suppression and HIV infection, OC increases the risk for AIDS-related morbidity and mortality [3] since HIV-infected individuals are known to have higher fungal loads than HIV-naïve patients [4]. Antifungal agents and combined anti-retroviral therapy (cART) are standard care at most HIV clinics. Prolonged antibiotic and antifungal therapy and prophylaxis are challenging in resource-limited countries, where systemic resistant infections are costly to treat and often result in increased patient morbidity and mortality [5].

Among the most commonly prescribed antifungal agents are the azoles (fluconazole, itraconazole, ketoconazole, voriconazole), echinocandins (anidulafungin, caspofungin, micafungin) and polyenes (nystatin, amphotericin B). Oral fluconazole is the treatment of choice. Patients tolerate fluconazole better than itraconazole and ketoconazole [6] and even though topical therapy (clotrimazole, nystatin and miconazole) may adequately treat initial episodes of oropharyngeal candidiasis [7], fluconazole is found to be more effective.

*Candida* species express drug resistance and virulence-related proteins in their cell membranes, which are pivotal in fungal pathogenesis. Virulence is mediated through morphogenesis and invasion [8]. Cell membrane proteins are involved in drug modification, detoxification, and resistance in both prokaryotic and eukaryotic systems [9]. The following pluripotent proteins are response mechanisms to stress that enable survival: **i)** ATP-binding cassette transporters Cdr1p and Cdr2p which act as efflux pumps to expel azoles and other drugs in *C. albicans* [10],[11] and elicit resistance, **ii)** Mdr1p, an efflux pump transporter implicated in *C. albicans* and *C. dubliniensis* azole resistance [12],[13], **iii)** Heat shock protein Hsp90, a chaperone that binds to human salivary peptides to mediate fungicidal activity and is implicated in antifungal drug resistance [14],[15], **iv)** Hsp70 proteins SSA1 and SSA2, which affect the fungicidal activity of human antimicrobial peptides [16]. SSA1 is associated with disseminated oropharyngeal disease [17], **v)** NADPH dehydrogenase EBP1 (estrogen binding protein 1) which binds with high affinity to mammalian estrogen [18], **vi)** pH-responsive protein 2 (PHR2), which may also be involved in pathogenesis [19] and **vii)** ECM33, an adhesin implicated in caspofungin resistance [20]. Additionally, lanosterol 14-alpha demethylase (Erg11p) catalyses C14-demethylation of lanosterol, a critical step for ergosterol biosynthesis [21]. Its involvement in the subpathway that synthesizes zymosterol from lanosterol is affected by the action of azole drugs, which bind to Erg11p and reduce ergosterol production in *Candida*. Twenty-five other enzymatic reactions take place in this biosynthetic pathway, with Erg1p, Erg2p and Erg24 being used as targets by other antifungal drugs [22]. Upregulation of Erg11p expression has been associated with increased resistance to azole in *C. albicans* [23], *C. tropicalis* [24] and *C. auris* [25].

Mechanistic studies of these membrane proteins are critical to the development of novel therapies to combat azole resistance in *Candida*, particularly in immunocompromised patients. Therefore, the objective of this study was to elucidate the mechanisms of drug-resistance-related *Candida* proteins detected in two cohorts of African HIV-infected individuals.

## 2. Materials and methods

### 2.1. Sampling

Ethics approval was obtained from the Research Ethics Committee of the University of the Western Cape in Cape Town, South Africa and from the Bamenda Regional Hospital Institutional Review Board (IRB) in the North West Province of Cameroon under ethics protocol numbers 07/2/40 and R.005/MPH.RDPH.RHB/359. The study protocol followed in accordance with the revised Declaration of Helsinki [26] and written informed consent was obtained from all participants for both the collection and storage of samples for future analyses. The study included HIV-positive patients presenting with white pseudomembranous plaque on the tongue or other visible oral candidiasis. Patients who received antifungals or other antimicrobial medications within two weeks prior to sample collection were excluded from the study. Oral swabs were collected for laboratory processing from 20 study participants recruited from health clinics in the Cape Flats region in Cape Town, South Africa and a further 20 recruited from Bamenda, Cameroon.

### 2.2. Identification and antifungal drug susceptibility testing of Candida isolates

Swab samples were plated onto Sabouraud agar (Cat. no. 84088; Sigma-Aldrich, St. Louis, MI, USA) and incubated at 37 °C for 24 hours. Presumptive species identification was achieved by 24–72 hour culture on Fluka chromogenic *Candida* identification agar (Cat. no. 94382; Sigma-Aldrich, St. Louis, MI, USA) and Oxoid chromogenic *Candida* agar (Cat. no. CM1002A; Oxoid, Hampshire, UK), incubated at 30 °C and species identification was confirmed using Gram stain morphology, growth on selective media, the germ tube test and API ID 32 C biochemical testing (Cat. no. 32200; bioMérieux, Marcy l'Etoile, France). Drug susceptibility was tested against the azoles (fluconazole, itraconazole, posaconazole and voriconazole), echinocandins (anidulafungin, caspofungin and micafungin), amphotericin B and 5-flucytosine using a Clinical and Laboratory Standards Institute (CLSI) approved broth microdilution susceptibility platform, as previously described [27].

### 2.3. Isolation of Candida cell membrane proteins

Drug resistance-related protein profiles expressed by different *Candida* species regarded as susceptible or resistant to azoles (as determined by CLSI guidelines) were compared, along with the proteins expressed using other antifungal drug classes.

Isolates were cultured on Sabouraud agar plates for 24 hours at 37 °C, before individual colonies were picked and incubated in yeast extract peptone dextrose (YPD) broth (peptone 10 g/L and dextrose 40 g/L distilled water) at 37 °C for 16 hours with agitation. Culture density was measured at 600 nm absorbance and the biomass was recovered by centrifugation at 3000 g. The fungal cell pellet was washed in 2 mL of sterile distilled water before being re-pelleted at 3000 g for 10 minutes. The isolated pellet was then resuspended in 2 mL homogenizing buffer with the protease inhibitor phenylmethylsulfonyl fluoride (PMSF) (50 mM Tris-HCl, pH 7.5, 2 mM EDTA, 1 mM PMSF), using a protein isolation method based on the one described by Niimi *et al* [10]. One-millimetre borosilicate glass beads (Cat. no. Z273619, Sigma-Aldrich, USA) were used to disrupt the fungal cells, by placing the tubes in a vortex for 6 minutes. The cell debris were pelleted at 5000 g for 10 min at 4 °C and the lysates (containing fungal cell membrane components) were then pelleted at 20 000 g for 1 hour at 4 °C, to result in a crude membrane fraction.

Protein concentration was determined by the Bradford method [28], using a Bio-Rad Bovine Serum Albumin (BSA) Standards Set (Cat. no. 500-0207, Bio-Rad, USA), according to the manufacturer's instructions. Sixteen microliters of a 10 mM Tris-HCl, pH 7.0, 5 mM EDTA solution were added to the isolated pellets, with standardized sample concentrations being subsequently diluted to an approximate protein concentration of 0.65 mg/mL.

**Figure 1.**
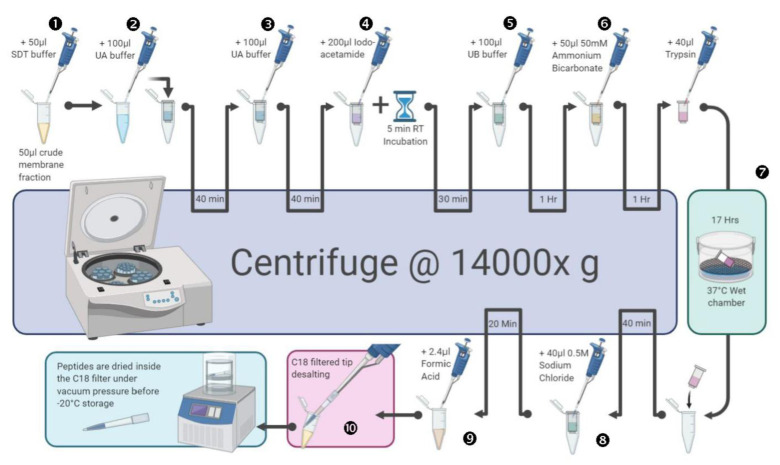
Filter-Aided Sample Preparation (FASP) overview. Membrane protein denaturing and binding to the Amicon filter takes place in steps ❶ to ❻: The SDS component of the SDT buffer assists in solubilising the membrane proteins in the crude extract, while the DTT acts as a reducing agent, breaking disulphide residue bonds. Membrane proteins are further denatured with the high urea content of the UA and UB buffers that further destabilise internal protein bonds. Iodoacetamide is a sulfhydryl-reactive alkylating reagent that blocks binding to the reduced cysteine residues and prevents disulphide bonding and protein refolding while the addition of bicarbonate renders the proteins in a highly-charged state, improving Amicon filter bonding. The addition of Trypsin and the overnight incubation at 37 °C in step ❼, further denatures the membrane proteins that are eluted from the Amicon filter by the interaction between the positively-charged proteins and negatively-charged chloride ions in the sodium chloride solution used in step ❽. Formic Acid further cleaves the membrane proteins into peptides at their C- or N- terminal domains (step ❾) before the eluted proteins then bind to a derivatised C18 filter in the StageTip (step ❿) through hydrophobic interactions. This step allows for other salts, buffers and chaotropes to be eluted. The tip with the bound proteins is then dried under vacuum pressure and stored at -20 °C until needed for HPLC-MS. This figure was created by an author (RF) using BioRender (https://biorender.com/).

Using filter-aided sample preparation (FASP) on the cell fractions, 50 µL samples were mixed 1:1 with SDT lysis buffer (4% SDS, 100 mM Tris-HCl pH 7.6, 0.1 M DTT), then mixed with an equal volume of UA buffer (8 M urea, 100 mM Tris-HCl, pH 8.5) and concentrated on an Amicon ultra 10 kDa MWCO filter (EMD Millipore, USA) by 40 minutes centrifugation at 14000 g. For simplicity, all subsequent centrifugation steps were performed at 14000 g. Next, 200 µL UA buffer was added and the samples were again centrifuged for 40 minutes before isolated proteins were alkylated with the addition of 100 µL of 0.05 M iodoacetamide in UA buffer. After a 5-minute incubation and 30-minute centrifugation, 100 µL of UB buffer (8 M urea, 0.1 M Tris-HCl pH 8.0) was added. This was followed by another hour centrifugation, the addition of 100 µL 50 mM ammonium bicarbonate solution before a 1-hour centrifugation cycle. Next, 40 µL trypsin was added and the filter was incubated at 37 °C for 17 hours in a wet chamber. The filter was subsequently transferred to a new tube and centrifuged for 40 minutes, followed by the addition of 40 µL of a 0.5 M sodium chloride solution and another 20-minute centrifugation cycle. Finally, the solution was acidified by the addition of 2.4 µL formic acid solution. The filtrate was then desalted using C18 StageTips (Thermo-Fisher Scientific, USA) according to the manufacturer's instructions, before drying *in vacuo* and -20 °C storage (Figure 1). Dried peptides were dissolved in 5% acetonitrile in 0.1% formic acid and 10 µL injections were made for nano-LC chromatography.

### 2.4. HPLC-MS protein identification

Mass spectrometry experiments were performed on a Thermo Scientific EASY-nLC II connected to a LTQ Orbitrap Velos mass spectrometer (Thermo Scientific, Bremen, Germany) equipped with a nano-electrospray source. For liquid chromatography, separation was performed on an EASY-Column (2 cm, ID 100 µm, 5 µm, C18) pre-column followed by XBridge BEH130 NanoEase (15 cm, ID 75 µm, 3.5 µm, C18) column with a flow rate of 300 nL/min. The gradient used was from 5–17% B in 5 min, 17–25% B in 90 min, 25–60% B in 10 min, 60–80% B in 5 min and kept at 80% B for 10 min. Solvent A was 100% water in 0.1% formic acid, and solvent B was 100% acetonitrile in 0.1% formic acid.

The mass spectrometer was operated in data-dependent mode, to automatically switch between Orbitrap-MS and LTQ-MS/MS acquisition and data acquired using the Xcalibur software package (Thermo Scientific Cat. No. OPTON-30487). The 20 most intense ions were isolated and fragmented in a linear ion trap (number of accumulated ions 1.5 × 10^4^) using collision induced dissociation. The lock mass option (polydimethylcyclosiloxane; *m/z* 445.120025) enabled accurate mass measurement in both the MS and MS/MS modes.

Thermo Proteome Discoverer 1.3 software (Thermo Scientific, Bremen, Germany) was used to identify proteins via automated database searching (Mascot, Matrix Science, London, UK, and Sequest) of all tandem mass spectra against the Uniprot database [29], with the aim of exclusively identifying specific protein functions related to antifungal drug resistance and proteins that affected the organism's pathogenicity and virulence. Proteins were considered positively identified when they were characterised with at least 1 tryptic peptide per protein, a Mascot score threshold of 20 and Sequest score threshold of 1.5. In order to include only significant data, only proteins with a mascot score above 24 were included in the study.

## 3. Results

### 3.1. Cohort demographics and combination ART

The majority of participants were female (80%) with a median age of 33.5 years (age range 24 to 70). Recruits were on the following ART at the time of sample collection: 82.5% receiving Lamivudine, 52% on Nevirapine, 42.5% on Zidovudine, 37.5% on Stavudine, 27.5% receiving Efavirenz, one person (2.5%) received Tenofovir and one person received Lopinavir/ritonavir as part of their 1st line cART regimen. Of the 40 patients, seven (17.5%) were confirmed HIV-positive but planned to begin cART, pending viral load testing results.

### 3.2. Drug resistance protein profiles

Standardized cell membrane fractions from seven *Candida* species (*C. albicans*, *C. dubliniensis*, *C. glabrata*, *C. tropicalis*, *C. krusei*, *C. rugosa* and *C. parapsilosis*) expressing different azole resistance patterns, were prepared for High Performance Liquid Chromatography-Mass Spectrometry (HPLC-MS) analysis.

**Figure 2.**
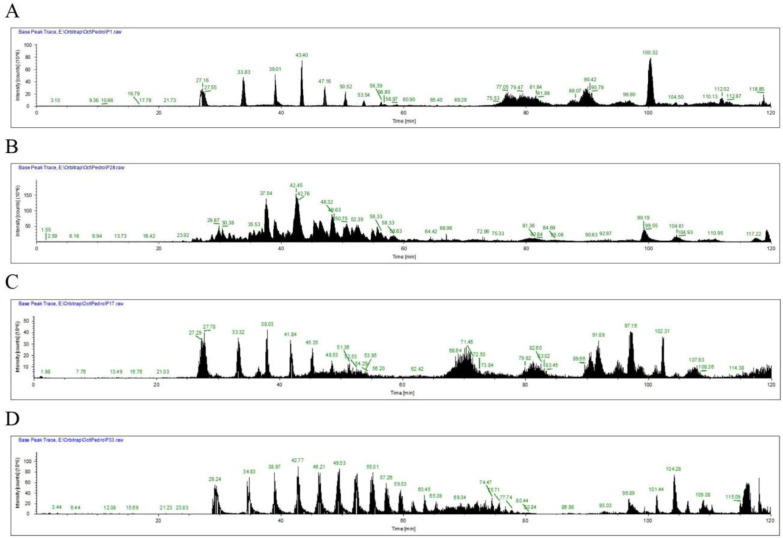
Chromatograms for (A) FCZ-susceptible *C. albicans*, isolate SA201; (B) FCZ-resistant *C. albicans*, isolate C199; (C) FCZ-intermediate resistant *C. glabrata*, isolate SA92; (D) FCZ-resistant *C. krusei*, isolate C144.

Chromatograms obtained after HPLC-MS analysis differentiated between *Candida* species as well as between fluconazole-susceptible and fluconazole-resistant strains of the same species (Figure 2A–D ).

### 3.3. Expression of azole resistance-related proteins

Various colonization and resistance mechanisms detected in azole-resistant isolates were elucidated using HPLC-MS. Representatives of heat-shock proteins were detected in all isolates. The identified heat shock proteins included Hsp70 (SSA1) and Hsp90 (expressed by all species). Hsp70 (SSA1 and SSA2) and Hsp90 were found in resistant isolates across all seven *Candida* species in our study, with Hsp70 (SSA1) expressed in seven of the nine *C. glabrata* strains tested. Of these seven, six Hsp70 (SSA1) positive isolates were also resistant to at least one azole drug tested. Only a single *C. glabrata* isolate was susceptible to all tested drug classes and did not express Hsp70, highlighting the possible pluripotent nature of this protein. In the *C. glabrata* isolate that displayed both fluconazole and voriconazole resistance, Hsp70 (SSA1) (UniProtKB accession number P10591) was expressed with the highest mascot score (1835.96) and had a high MS sequence coverage of 42.37%. Hsp70 (SSA1) was also identified in the membrane fractions of *C. glabrata* isolates that were susceptible to all azoles tested but were resistant to amphotericin B (one isolate) or had intermediate resistance to micafungin (one isolate). Heat-shock protein Hsp70 (SSA2) was expressed by resistant representatives of all species with the exception of *C. dubliniensis*. Other resistance-related proteins included NADPH dehydrogenase oxidoreductase EBP1 (expressed by *C. albicans*) and PHR2 (expressed by *C. albicans* and *C. dubliniensis* resistant isolates). The expression of a multidrug resistance transporter protein Cdr1p was seen in two *C. albicans* azole-resistant fractions, with multidrug efflux transporter Cdr2p being present in a *C. albicans* isolate resistant to all four azole drugs tested. Transporter protein Cdr4p was also identified in eight *C. albicans* resistant fractions (Table 1). Proteins associated with ergosterol biosynthesis were found in *C. albicans*, *C. dubliniensis*, *C. glabrata* and *C. tropicalis* cell fractions. Although Erg11p was the most predominant protein in this group, other proteins that are involved in ergosterol biosynthesis were also identified. They included Erg9p (which synthesises squalene from farnesyl diphosphate), Erg1p (which synthesises squalene epoxide from squalene), Erg6p (which synthesises fecosterol from 4,4 dimethylzymosterol) and Erg3p (which synthesises ergosta-5,7,24(28)-trienol from episterol). A *C. albicans* isolate highly resistant to all tested azole drugs had the highest detected mascot score for Erg11p (188.9), while also expressing Erg6p. Some proteins appeared to be more prevalent in certain species, for example, efflux pumps, corticosteroid and estrogen binding proteins were only present in *C. albicans* isolates, while other protein types were more widely distributed (Table 1).

While most isolates initially selected for their susceptibility to azoles were also susceptible to other drug classes, one *C. glabrata* isolate and the sole *C. rugosa* representative expressed resistance to amphotericin B. In the latter, only salivary histatin binding heat-shock proteins Hsp70 (SSA1/2) and Hsp90 were identified along with PHR2.

**Table 1.** Individual drug-resistance and pathogenicity-related proteins seen in azole-susceptible and -resistant isolates, identified by HPLC-MS and the UniProt database.

***Candida* species**	**Azole resistance**	**ABC transporters**			**Increased pathogenicity**	**Estrogen binding**	**Heat shock proteins**			**Ergosterol biosynthesis proteins**				
		Cdr1p	Cdr2p	Cdr4p	pH-responsive protein 2	EBP1	Hsp70		Hsp90	Erg11p	Erg1p	Erg3p	Erg6p	Erg9p
							SSA1	SSA2						
**UniProtKB accession numbers**		P43071	P78595	O74676	O13318	P43084	P41797	P46587	P46598	P10613; P14263; P50859	O13306	O93875; P50860	O74198; Q6FRZ7; Q875K1	P78589; Q9HGZ6
*C. albicans*	S (n = 10)	√ (3)	_	√ (7)	√ (9)	√ (4)	√ (10)	√ (9)	√ (10)	√ (3)	_	_	√ (3)	√ (1)
	R (n = 10)	√ (2)	√ (1)	√ (8)	√ (9)	√ (3)	√ (10)	√ (5)	√ (7)	√ (5)	_	√ (1)	√ (1)	_
*C. glabrata*	S (n = 3)				√ (1)	_	√ (2)	√ (2)	√ (2)	_	_	_	√ (1)	_
	R (n = 6)				_	_	√ (5)	√ (3)	√ (5)	√ (3)	_	√ (1)	√ (1)	√ (3)
*C. dubliniensis*	S (n = 3)				√ (2)	_	√ (3)	√ (2)	√ (3)	√ (1)	√ (1)	_	_	_
	R (n = 1)				√ (1)	_	√ (1)	_	√ (1)	_	_	_	_	_
*C. krusei*	S (n = 1)				_	_	_	_	√ (1)	_	_	_	_	_
	R (n = 2)				_	_	√ (1)	√ (1)	√ (1)	_	_	_	_	_
*C. tropicalis*	R (n = 2)				_	_	√ (2)	√ (2)	√ (1)	√ (2)	_	_	√ (1)	_
*C. rugosa*	S (n = 1)				_	_	√ (1)	√ (1)	√ (1)	_	_	_	_	_
*C. parapsilosis*	S (n = 1)				√ (1)	_	√ (1)	√ (1)	√ (1)	_	_	_	_	_

√ : protein present; -: protein not present

### 3.4. Expression of cross-resistance-related proteins

Isolates demonstrating cross-resistance to both azoles and other drug classes expressed PHR2, a protein family associated with increased pathogenicity. PHR2 was seen in multi-drug resistant *C. dubliniensis* and in three *C. albicans* isolates where one expressed cross-resistance to amphotericin B and two expressed cross-resistance to 5-flucytosine. Interestingly, one of the *C. albicans* azole-resistant isolates resistant to 5-flucytosine, expressed 9 resistance-associated proteins including two multi-drug transporters, three distinct heat-shock proteins, NADPH dehydrogenase (EBP1), PHR2 and two ergosterol biosynthesis proteins. Incidentally, this was the isolate expressing the highest mascot score for Erg11p. Another *C. albicans* isolate with resistance to both azoles and 5-flucytosine expressed a similar protein profile, with the exception of Hsp70 (SSA2) and NADPH dehydrogenase.

### 3.5. Comparison of resistance protein profiles of patients on cART and those not yet on cART

Of the patients who were not yet on cART at the time of sample collection, five were colonised by *C. albicans* (three of which were highly resistant to fluconazole and two of these also being resistant to 5-flucytosine), one was colonised by a multi-drug resistant (MDR) *C. dubliniensis* isolate and one was colonised by *C. glabrata*. When comparing the protein profiles between patients who were yet to start cART and those already on cART, it was noted that apart from the presence of the ubiquitous heat shock proteins, the species isolated from patients not on cART also expressed pH-responsive protein 2 (seen in the MDR *C. dubliniensis* and four *C. albicans* isolates), while one of the Cdr1p and two Cdr4p efflux transporters were identified in two *C. albicans* isolates from this group. Ergosterol biosynthesis proteins were identified in a *C. albicans* isolate and in the MDR *C. dubliniensis* isolate (Erg9p, Erg11p and Erg3p) belonging to this group.

## 4. Discussion

Our previous finding of the high resistance of *Candida* to various antifungal drugs seen in these HIV patients [27] is supported by this attempt to elucidate the different mechanisms that increase the pathogenicity and drug resistance of these organisms. This study focused on 40 novel *Candida* isolates whose membrane protein composition were characterised to better understand their role in drug resistance.

*Candida* species can express estrogen-and progesterone-binding proteins, explaining the higher predisposition of females to candidiasis [30],[31] and in the current study, estrogen-binding proteins were identified in *C. albicans* cell fractions in the form of EBP1.

The heat-shock proteins Hsp70 (SSA1 and SSA2) and Hsp90 are known to bind to HTN3/histatin-5 found in saliva, affecting the fungicidal activity of these native antimicrobial proteins [14],[15],[32] and leading to resistance [33] and increased oral *Candida* colonization [34], especially in immunocompromised patients [35]. Histatins are small (7–38 amino acids) histidine-rich, broad spectrum, cationic immunological peptides that act on the cell membrane causing pore formation and fungal cell lysis. Demonstrating potent antifungal activity, histatins are known to interact with Hsp70 (SSA1/2) and kill *Candida* [32]. Hsp70 (SSA1) was previously reported to be upregulated in fluconazole-resistant membrane fractions of *C. glabrata* [36], while in contrast, it was found by the same group to be downregulated in voriconazole-resistant isolates [37]. The presence of Hsp70 (SSA1) in most tested isolates, the ability of this protein to affect the fungicidal activity of the host's antimicrobial peptides [16] and its association with oropharyngeal disease [17] are factors that can directly influence the treatment outcomes of HIV patients.The ubiquitous Hsp90, on the other hand, has a role in supporting cell survival by stabilizing enzymes during stress [13], but has also been implicated in the rapid acquisition [38] and increased fluconazole resistance in *Candida* biofilms [15].

Multidrug resistance proteins were detected in most *C. albicans* cell membrane fractions tested in this study. The appearance of multidrug resistance protein Cdr1p may lead to resistance to a range of compounds as well as to azole antifungal drugs [39]. Cdr1p plays a role in fluconazole drug resistance [40] and confers resistance to cycloheximide, chloramphenicol and miconazole [41]. The up-regulation of Cdr1p in the cell membrane of a *C. glabrata* fluconazole-resistant strain [42] and in azole-resistant *C. parapsilosis* [43] have previously been documented, but this protein was not detected in membrane fractions of azole resistant *C. glabrata* in the present study. The sole *C. parapsilosis* isolate tested in this study was a highly susceptible isolate, possibly explaining the lack of this drug resistance mechanism on this strain. Cdr2p (identified in a *C. albicans* isolate resistant to all four azole drugs tested) has the ability to not only confer resistance to azoles but also to other antifungals such as terbinafine and amorolfine in addition to metabolic inhibitors [44]. The ability of histatin-5 to inhibit the development of Cdr1p and Cdr2p-mediated multidrug resistance [45], demonstrates how various cellular mechanisms may act together to modulate fungal pathogenicity. The identification of Cdr4p in eight drug-resistant *C. albicans* cell membrane fractions deserves attention since Cdr4p is an ABC transporter protein involved in azole resistance in filamentous fungi [46].

Five of the 25 proteins involved in ergosterol biosynthesis were found in this study. Azole drugs target this biosynthesis pathway by inhibiting Erg11p, leading to a block in ergosterol synthesis and the accumulation of toxic sterol intermediates by Erg6p and Erg3p [22]. *Candida* is known to mutate the Erg11 gene [23],[47] and demonstrate drug target overexpression of Erg11p [48] as part of its resistance mechanisms against azole drugs.

Coupled with filter-aided sample preparation, HPLC is a useful method for demonstrating antifungal susceptibility profiles and, with further developments in sample preparation, could become a rapid, reliable and practical technique for the early detection of resistance and subsequent prompt treatment of resistant *Candida* infections. HPLC analysis can be completed within hours, favouring its application over the traditional time-consuming processes of culture and disc diffusion or microdilution methods for drug susceptibility testing which may take days to complete. Comparatively, the cost, equipment specifications and skilled operators needed to perform FASP-HPLC analysis outweigh those associated with traditional drug resistance testing methods, not to mention the higher fungal biomass needs in relation to more sensitive molecular sequencing methods.

Although the introduction of cART has vastly improved the quality of life for the HIV-infected, it has minimally impacted the observed number of *Candida* infections [49],[50]. The large number of immunocompromised HIV-positive patients in Africa receiving non-protease-inhibitor-based first line cART [51] and the prevalence of multidrug-resistant candidiasis, suggests a bleak future, barring any natural therapeutic intervention without adverse cART interactions [52]. A limitation of this study is that since only seven of the 40 isolates were collected before the commencement of cART, an association between the effect of cART on protein expression could not adequately be compared, thus creating an avenue which may be further explored.

Furthermore, the inability to significantly differentiate between individual protein expression profiles and their relation to specific antifungal classes in MDR isolates indicates that further optimization of the protein isolation methods should be prioritized in future studies. In spite of this methodological limitation, it was possible to identify the presence of protein profiles in organisms expressing specific drug resistance patterns within the different species studied. The combination of diverse drug resistance and survival mechanisms were notably more prevalent in *C. albicans*, *C. dubliniensis* and the inherently fluconazole-resistant *C. glabrata*. These isolates expressed proteins that enhance their fungicidal activity by increasing their adherence to salivary peptides and improving the binding of fungal proteins to mammalian hormones, modifications that are instrumental in the ability of these organisms to colonise immunocompromised patients and resist antifungal drugs.

The possibility exists that cell membrane changes could have occurred in response to environmental stress, such as pH changes during protein isolation, rather than to stress induced by drugs. Additionally, given that most UniProt strains are isolates from other geographical regions, and since there is a paucity of studies on isolates from Africa, it is possible that the isolates have sequence polymorphisms and different amino acid sequences when compared to other previously tested populations. In essence, the limited MS data for *Candida* isolates from sub-Saharan Africa identifies the lack of proteomics-based drug resistance monitoring in this study's sample populations.

## 5. Conclusions

Fluconazole-resistance is steeply rising, and it is the most frequently prescribed antifungal for HIV-associated opportunistic infections. Antifungals such as echinocandins, are usually considered for treating patients who show resistance due to previous azole exposure, or when *C. glabrata, C. parapsilosis* or *C. krusei* have been identified [53]. Although shown to be as effective in treating OC with less serious side effects, echinocandins have a greater relapse rate, contributing significantly to ever-increasing antifungal resistance prevalence [54],[55]. Amphotericin B is used when patients show resistance to fluconazole and echinocandins. However, in very ill patients, it may prove toxic [56] and has limited availability in resource-poor countries due to its cost.

Reported drug interactions between systemic antifungals (particularly azoles) and antiretrovirals have led to the addition of special guidelines for their use with combination ART for HIV-infected persons by the US Department of Health and Human Services [57]. Moreover, the evolving drug resistance and subsequent circulation of multidrug-resistant or inherently drug-resistant, non-albicans species in the treatment-naïve population not only limits treatment options but may also reduce the tolerance of combination ART through drug interactions.

Thus, there is an urgent need for the development of novel, non-toxic, broad spectrum, highly potent, natural antifungal therapies. HPLC provides beneficial protein abundance data that is essential for understanding the role of individual proteins in drug resistance mechanisms *in vitro*, thus establishing a role in studies contributing to the development of novel therapies.
